# Multi-omics signatures of diverse plant callus cultures

**DOI:** 10.5511/plantbiotechnology.24.0719a

**Published:** 2024-09-25

**Authors:** June-Sik Kim, Muneo Sato, Mikiko Kojima, Muchamad Imam Asrori, Yukiko Uehara-Yamaguchi, Yumiko Takebayashi, Thi Nhung Do, Thi Yen Do, Kieu Oanh Nguyen Thi, Hitoshi Sakakibara, Keiichi Mochida, Shijiro Ogita, Masami Yokota Hirai

**Affiliations:** 1 RIKEN Center for Sustainable Resource Science, Yokohama, Kanagawa 230-0045, Japan; 2 Institute of Plant Science and Resources, Okayama University, Kurashiki, Okayama 710-0046, Japan; 3 Program in Biological System Science, Graduate School of Comprehensive Scientific Research, Hiroshima Prefectural University, Shobara, Hiroshima 727-0023, Japan; 4 Department of Life Sciences, University of Science and Technology of Hanoi, Vietnam Academy of Science and Technology, 18 Hoang Quoc Viet, Cau Giay, Hanoi, Vietnam; 5 Graduate School of Bioagricultural Sciences, Nagoya University, Nagoya, Aichi 464-8601, Japan; 6 RIKEN Baton Zone Program, Yokohama, Kanagawa 230-0045, Japan; 7 School of Information and Data Sciences, Nagasaki University, Nagasaki, Nagasaki 852-8521, Japan; 8 Kihara Institute for Biological Research, Yokohama City University, Yokohama, Kanagawa 244-0813, Japan

**Keywords:** callus culture, hormonome, transcriptome, widely targeted metabolome

## Abstract

Callus cultures are fundamental for plant propagation, genetic transformation, and emerging biotechnological applications that use cellular factories to produce high-value metabolites like plant-based drugs. These applications exploit the diverse metabolic capabilities of various plant species. However, optimizing culture conditions for specific applications necessitates a deep understanding of the transcriptome, metabolome, and phytohormone profiles of different species. Comprehensive comparative studies of callus characteristics across species are limited. Here, we analyzed the transcriptome, metabolome, and phytohormone profiles of callus cultures from tobacco (*Nicotiana tabacum*), rice (*Oryza sativa*), and two bamboo species (*Phyllostachys nigra* and *P. bambusoides*). Multivariate analyses of metabolome data revealed similar metabolic trends in these diverse callus cultures and identified metabolites that differ between species. Hormone profiling showed distinct species-specific patterns and notable cytokinin diversity, even between the bamboo species. Moreover, a comparative analysis of 8,256 pairs of syntenic genes between rice and bamboo revealed that 84.7% of these orthologs showed differential expression, indicating significant transcriptomic diversity despite phylogenomic relatedness. Transcriptional regulation of developing organs often involves conserved gene expression patterns across species; however, our findings suggest that callus formation may relax evolutionary constraints on these regulatory programs. These results illustrate the molecular diversity in callus cultures from multiple plant species, emphasizing the need to map this variability comprehensively to fully exploit the biotechnological potential of plant callus cultures.

Plant callus cultures have long been used in plant biotechnology. Their totipotency and ability to proliferate make them versatile platforms for various applications, including tissue culture for propagation, genetic transformation for molecular genetics and crop breeding (Chen et al. 2022; Efferth 2019), and as models for studying development and redifferentiation in plants (Morinaka et al. 2023; Zhai and Xu 2021). Recent applications include the use of callus cultures in metabolic engineering, where they are being explored for use as cell factories for producing valuable metabolites. Indeed, plants have remarkable abilities to produce a wide variety of metabolites, including high-value compounds such as anti-cancer drugs, and these applications can leverage the diverse metabolic capabilities inherent in different plant species (Hashem et al. 2022; Ono and Murata 2023).

Leveraging these capabilities will require a deep understanding of the intrinsic characteristics and variability of callus cultures across different plant species and this understanding remains limited. Moreover, a deep understanding will require the exploration of diversity and commonality using multiple omics datasets and diverse plant species. For example, comparing orthologous genes in transcriptome data may reveal conserved regulatory mechanisms underpinning the development of common organs such as leaves (Vercruysse et al. 2020). Cross-species comparisons of metabolome data may identify unique metabolic networks and chemical markers that distinguish closely related plant species (Shen et al. 2023). Similarly, profiling phytohormones, including active forms, inactive forms, precursors, and derivatives may illustrate the diversity of regulatory networks underpinning various biological processes in plants (Široká et al. 2022). Therefore, comparing transcriptome, metabolome, and hormonome data from callus cultures across diverse plant species may help fill the gap in our understanding of the intrinsic characteristics of this distinct tissue type and enable multiple biotechnological applications.

To compare callus cultures, we analyzed transcriptome, metabolome, and phytohormone profiles of callus cultures induced from tobacco (*Nicotiana tabacum* L. cv. SR1 [SR]), rice (*Oryza sativa* L. cv. Nipponbare [Os]), and two bamboo species (*Phyllostachys nigra* (Lodd. ex Lindl.) Munro var. Henonis [Pn] and *P. bambusoides* Siebold and Zucc. [Pb]) (Supplementary Text) (Kim et al. 2024). These data were collected over four weeks under conditions of continuous high humidity or gradually decreasing humidity with airflow. The transcriptome data can be accessed from the DNA Data Bank of Japan BioProjects with the following accessions; PRJDB16707 (SR), PRJDB16708 (Os), PRJDB16736 (Pn), and PRJDB16709 (Pb). The metabolome and phytohormone profile data are available from DROP Met (https://prime.psc.riken.jp/menta.cgi/prime/drop_index) with accession number DM0060.

For transcriptome analysis of the bamboo species, the genome sequence of the diploid Moso bamboo (*P. edulis*) was used as a reference (Zhao et al. 2018), which did not account for the polyploidy or other specific genomic characteristics of Pn and Pb. Orthologous gene sets between species were established by using OrthoFinder version 2.5.5 (Emms and Kelly 2019). Genome-wide synteny of rice (IRGSP-1.0; Kawahara et al. 2013) and bamboo genes was evaluated by MCScan as a part of JCVI utility libraries version 1.2.10 (Tang et al. 2008).

To explore the chemical diversity of these four callus cultures, we conducted multivariate analyses of metabolome and phytohormone datasets. First, we applied partial least squares regression–discriminant analysis (PLS-DA) to reduce the dimensionality of the metabolome and phytohormone data using the mixOmics package (Rohart et al. 2017), which separated the samples by species (Figure 1A). We evaluated the classification error rates of our PLS-DA model using cross-validation. The combination of Component 1 and Component 2 significantly reduced the classification error rate when using centroid distance, achieving 0.055 in the metabolome dataset and 0.043 in the hormonome dataset, with five-fold cross-validation repeated 50 times. The callus cultures showed generally similar trends in metabolite abundance, but tobacco callus accumulated notable amounts of certain metabolites, such as uridine, caffeic acids, and betaine, distinguishing it from the other Poaceae-derived callus cultures (Supplementary Table S1).

**Figure 1:**
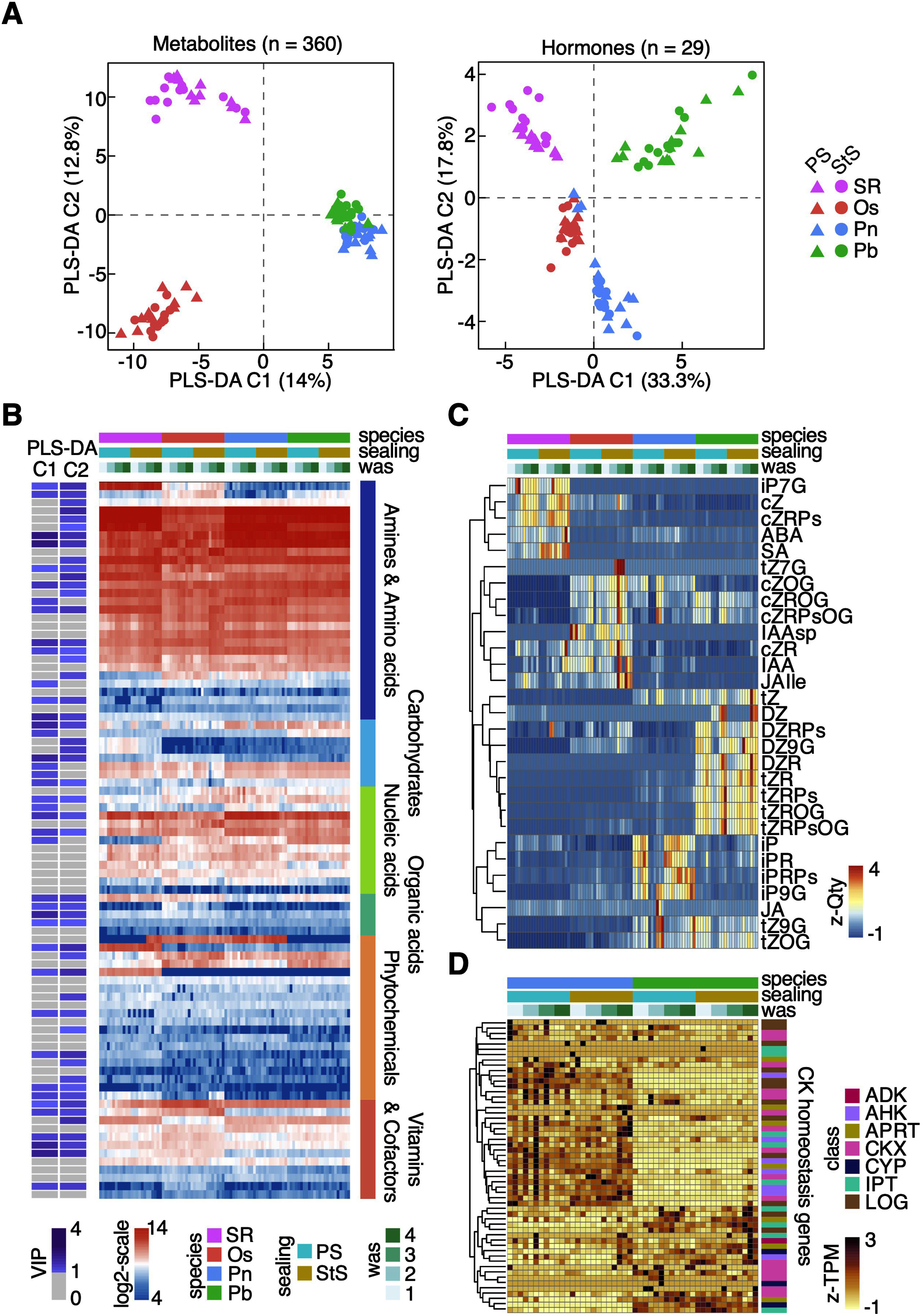
Figure 1. Metabolome and hormonome-based species-specific molecular signatures in plant callus cultures. A. Scatter plots of two PLS-DA components (C1 and C2) showing the separation of callus samples based on metabolome (left) and hormonome (right) data. Colors represent different plant species: SR (*N. tabacum*), Os (*O. sativa*), Pn (*P. nigra*), and Pb (*P. bambusoides*). Shapes indicate varied moisture conditions by different sealing types: PS (Parafilm Sealing; high humidity without airflow) or StS (Surgical tape Sealing; decreasing humidity with airflow). B. Heatmap displaying the log_2_-scaled abundance of 96 metabolites, classified into six categories based on KEGG BRITE information. Variable importance for prediction (VIP) scores of each metabolite for two PLS-DA components shown in (A) are presented in a color scale. The species, the sealing type, and the weeks-after-sealing (was) values for each sample are indicated by different colors, which are also used in C and D. C. Heatmap showing *z*-standardized quantities (z-Qty) of 29 hormones, indicating species-specific metabolite accumulation patterns with hierarchical clustering. D. Heatmap displaying the *z*-standardized digital expression levels (z-TPM) of cytokinin (CK) homeostasis genes, with hierarchical clustering illustrating species-specific expression patterns in Pn and Pb. Genes involved in CK signaling, biosynthesis, or metabolism are categorized by KEGG ORTHOLOGY. Detailed information for the genes and metabolites of the heatmaps (B–D) are available in Supplementary Tables S1–S4.

Based on the PLS-DA, we identified the metabolites that significantly contributed to the species-wise sample grouping using Variable Importance in Projection (VIP) scores, which indicate the contribution of each metabolite to the predictive power of the model, with higher values representing greater importance (Figure 1B; Supplementary Table S1). Additionally, although the samples included those from different humidity conditions due to variations in sealing types, we observed no clear association with the environmental differences (Figure 1B). Overall, our comparative analysis of the metabolome dataset demonstrated species-specific metabolic properties within broadly similar trends in metabolite abundance across these callus cultures.

Next, we analyzed the phytohormone profiles of the callus samples. Using PLS-DA to visualize the distribution of samples based on the results of 29 plant hormones produced a clearer species-wise grouping than in the metabolome-based analysis. In particular, this grouping distinguished the two bamboo species (Figure 1A; Supplementary Table S2). To evaluate hormone abundance and interspecies differences, we created a hierarchically clustered heatmap of the *z*-scored hormone profiles and found species-specific hormonal profiles (Figure 1C). Notably, among the cytokinins (CKs), when comparing the two bamboo species, Pn data exhibited a greater abundance of the *trans*-zeatin and dihydrozeatin types, while Pb data exhibited a greater abundance of the isopentenyladenine types (Figure 1C).

To measure the expression levels of genes involved in CK homeostasis, we compiled orthologous genes involved in cytokinin signaling, biosynthesis, and degradation in the bamboo genome to those of rice and *Arabidopsis thaliana* (Supplementary Table S3). The expression profiles of these CK-related genes also formed species-specific clusters for Pn and Pb, suggesting that these callus cultures also differ at the transcriptional level (Figure 1D; Supplementary Table S4). The diversity in CK side-chain structures could affect receptor affinity and stability in vivo, thus affecting various physiological processes in plants (Kiba et al. 2023; Sakakibara 2006). Although callus does not form organs, these differences in hormonal states might contribute to variability in responses to exogenous hormones and the potential for differentiation of the callus. Our comprehensive hormonal profiling of callus cultures from multiple plant species illustrates substantial numbers of species-specific differences, which may underpin variations in signaling pathways and physiological responses, suggesting a complex physiological landscape even in undifferentiated tissues in plants.

The transcriptome diversity we observed in cytokinin-related genes led us to examine transcriptome-wide species-specific differences. However, such cross-species transcriptome comparisons often face challenges in defining orthology between species due to the complex evolutionary history with gene duplications and losses. To address these challenges, we constructed orthologous relationships between genes in rice and bamboo based on the synteny of orthologous genes across Poaceae genomes (Tang et al. 2008). The genomes and deduced proteomes of rice (Nipponbare) and Moso bamboo identified syntenic blocks composed of 29,835 gene pairs (Figure 2A). A cross-species mapping of RNA sequencing (RNA-seq) reads from Pn and Pb callus to the Moso bamboo genome defined 8,256 single-gene pairs between rice and Moso bamboo for comparative transcriptome analysis (Figure 2B; Supplementary Table S5).

**Figure 2:**
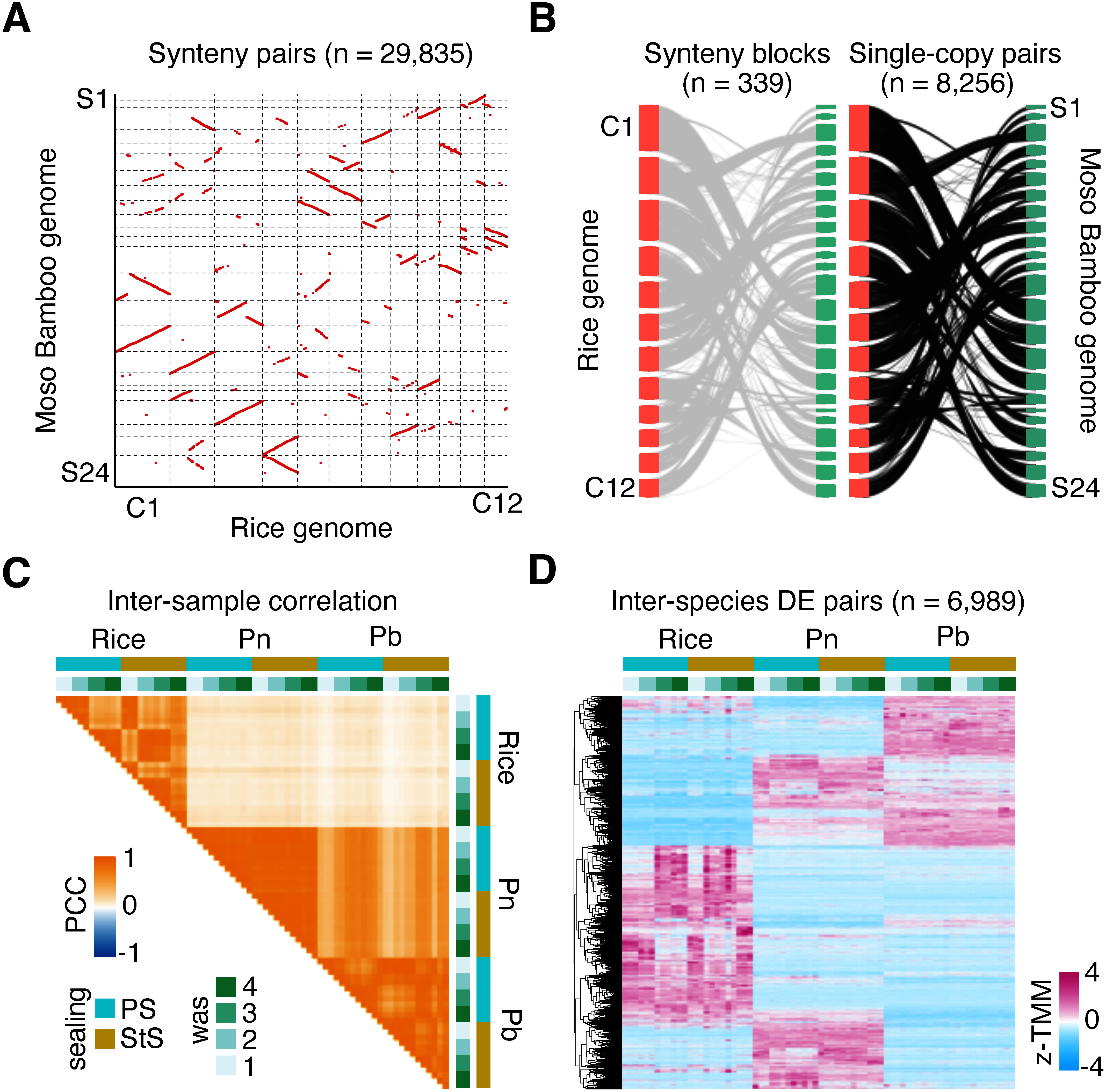
Figure 2. Cross-species transcriptome comparison between rice and two bamboo species. A. Scatter plot representing genome-wide synteny identified between chromosomes of rice (C1–C12) and Moso bamboo (S1–S24), comprising 29,835 gene pairs. Dotted lines border chromosomes. B. Schematic diagrams of rice and bamboo chromosomes illustrating syntenic relationships across chromosomes of synteny blocks (left) and single-copy gene pairs (right). C. Heatmap representing the Pearson’s correlation coefficient (PCC) of transcriptome data across different samples of rice, Pn, and Pb. The sealing type and the weeks-after-sealing (was) values for each sample are indicated by different colors. D. Heatmap presenting the *z*-standardized digital expression level with TMM normalization (z-TMM) of callus samples from rice, Pn, and Pb. The hierarchical clustering for species-specific expression patterns is presented. Detailed information for the genes of synteny is available in Supplementary Table S5.

For the gene expression analysis, we mapped RNA-seq reads to these reference genomes and compared expression levels. We normalized the data using transcripts per million (TPM) and trimmed mean of M values (TMM) methods, finding that TMM normalization provided better density plot consistency. Based on the TMM-normalized expression levels of the 8,256 genes, we evaluated transcriptome similarities among samples using correlation coefficients. This evaluation found no correlation between transcript levels in rice and the two bamboo species, but did find a correlation between transcript levels in the two bamboo species (Figure 2C). Using edgeR (Robinson et al. 2010), we selected differentially expressed genes (DEGs) for the pairs Os-Pn, Os-Pb, and Pn-Pb, detecting 5,896, 5,925, and 4,608 DEGs, respectively (false discovery rate [FDR] <0.01). The union of these DEGs accounted for 6,989 gene pairs, representing 84.7% of the 8,256 gene pairs, and their expression patterns exhibited clear species specificity (Figure 2D). While the number of DEGs might be exaggerated due to limited normalization strength for inter-genus comparison, the comparison of rice and bamboo transcriptomes demonstrated that they have highly diverse transcriptional profiles, with significant interspecies differences even within the same family (Poaceae) or genus (*Phyllostachys*).

This study highlights the molecular diversity of callus cultures across different plant species. Our widely-targeted metabolome analysis revealed similar trends in metabolite abundance associated with the callus state, along with distinct species-specific accumulation patterns for particular metabolites. The diversification of metabolomes, including specialized metabolites, is a central process in plant adaptation. Therefore, phylo-metabolomics or evolutionary metabolomics, which involves chemo-typing across evolutionarily diverse plants, would provide a valuable resource for leveraging such metabolic diversity in plants (Elser et al. 2023; Ono and Murata 2023). Moreover, our hormonome and transcriptome analyses further emphasized the species-specific characteristics of callus cultures in plants. The differences in hormonal states and transcriptional profiles among species suggest that even within the same family or genus, callus cultures exhibit substantial molecular diversity. For instance, we observed two bamboo species with distinct hormonal landscapes, each focusing on accumulating different cytokinin derivatives and exhibiting different expressions of the associated metabolic genes (Figure 1). This may reflect that even in the same *Phyllostachys* family, the significant phylogenetic distance between two bamboo species (Wang et al. 2021; Zhao et al. 2015) could substantially affect their metabolic preferences. Such molecular diversity may influence the effects of exogenous growth regulators in plant biotechnological applications, such as somatic embryogenesis and de novo organogenesis (Shin et al. 2020).

Callus cultures, being in an undifferentiated state, may lack the regulatory constraints and conserved signaling networks often observed in differentiated tissues across plant species (Wu et al. 2021). This may result in greater molecular differences between species, even in the same genus, and disordered cellular states depending on the originating species, organ, and induction processes. The diversified omics profiles we observed in plant callus cultures emphasize the importance of detailed mapping of the molecular variability in transcriptomes, metabolomes, and hormonomes, across plant callus cultures. Multi-omics approaches have enabled the identification of novel functional metabolites with critical roles in plant growth and development (Kawade et al. 2023). Such comprehensive mapping would enable researchers to unlock their full potential in biotechnological applications, including sustainable bioproduction of diverse metabolic compounds in plants (Li-Beisson et al. 2024), by ensuring the reproducibility, scalability, and environmental robustness of engineered callus cultures to improve their production yields.

## Abbreviations

CK: cytokinin
PCC: Pearson’s correlation coefficient
PLS-DA: partial least squares regression-discriminant analysis
PS: Parafilm Sealing
StS: Surgical tape Sealing
VIP: variable importance in projection

## Description of Supplementary Files

Supplementary Table S1. Details of the metabolites presented in Figure 1B.

Supplementary Table S2. Details of the phytohormones and their derivatives presented in Figure 1C.

Supplementary Table S3. Bamboo ortholog genes involved in cytokinin homeostasis.

Supplementary Table S4. Bamboo cytokinin biosynthesis/metabolism genes presented in Figure 1D.

Supplementary Table S5. Synteny-paring genes between rice and Moso bamboo genomes.

Supplementary Text. Culture conditions and subculture procedures for tobacco, rice, and bamboo calli.
