# Supplementary material for: Multi-omics signatures of diverse plant callus cultures
Source: Plant Biotechnol (Tokyo). 2024 Sep 25;41(3):309–14. doi: 10.5511/plantbiotechnology.24.0719a (PMC11921129; doi:10.5511/plantbiotechnology.24.0719a)
Supplement: Supplementary Data [file plantbiotechnology-41-3-24.0719a-s001.pdf]

Supplementary Method

Culture conditions for Tobacco calli

The tobacco calli used in this study were induced from leaf segments of an SR1 plant (*Nicotiana tabacum* L. cv. SR1 [SR]), using seeds provided by the Leaf Tobacco Research Center (Japan Tobacco, Tokyo, Japan). The SR calli were cultured on Linsmaier and Skoog medium (pH 5.7) containing 1 μM 2,4-D, 3% sucrose, and 0.3% gellan gum in sterilized Petri dishes with a diameter of 90 mm and a height of 15 mm. The SR calli cultures were maintained at 25°C with a 16-h light:8-h dark photoperiod, with light provided by fluorescent illumination (65 μmol m−2 s−1).

Culture conditions for rice and bamboo calli

The calli of rice (*Oryza sativa* L. cv. Nipponbare [Os]) and two bamboo species (*Phyllostachys nigra* (Lodd. ex Lindl.) Munro var. Henonis [Pn] and *P. bambusoides* Siebold and Zucc. [Pb]) were cultured on a modified Murashige and Skoog medium (pH 5.7) containing 10 μM picloram, 3% sucrose, and 0.3% gellan gum in sterilized Petri dishes with the same dimensions. The Os calli cultures were maintained at 25°C with a 16-h light:8-h dark photoperiod, with light provided by fluorescent illumination (65 μmol m−2 s−1), while the bamboo calli cultures were maintained at 25°C in the dark.

Subculture

The calli derived from these four species were routinely subcultured by transferring four pieces of callus, each approximately 500 mg in fresh weight and 10 mm in diameter, to a new Petri dish with fresh medium every four weeks. Each Petri dish was sealed with either parafilm (PS) or surgical tape (StS).
